# Epidemiology of hospital acquired urinary tract infections in a medical college hospital in Goa

**DOI:** 10.4103/0970-1591.45542

**Published:** 2009

**Authors:** Umesh S. Kamat, Agnelo Fereirra, Dilip Amonkar, Dilip D. Motghare, Manoj S. Kulkarni

**Affiliations:** Department of Preventive and Social Medicine, Goa Medical College, Bambolim, Goa, India; 1 Department of General Surgery, Goa Medical College, Bambolim, Goa, India

**Keywords:** Antimicrobial resistance, hospital acquired urinary tract infections, nosocomial infections

## Abstract

**Background:**

Hospital Acquired Urinary Tract Infection (HAUTI) is the commonest among the nosocomial infections, and hospital specific data concerning its magnitude and attributes is essential to its effective control.

**Materials and Methods:**

Prospective study was undertaken among 498 in-patients at the medical college hospital in Goa, employing the clinico-bacteriologic criteria of CDC, Atlanta, in the representative medicine and surgery wards. Antimicrobial sensitivity was tested using the Kirby-Bauer disc diffusion method.

**Statistical Analysis:**

Statistical significance of association was tested using the chi-square test and the unpaired t-test at 5% level of significance, while the strength of association was expressed as the Odd's ratio with 95% confidence interval calculated by Wolff's method.

**Results:**

While the overall infection rate was 8.03/100 admissions, 33.6% of the catheterized patients developed HAUTI. Effect of gender was found to remain restricted to the development of HAUTI among females at an earlier age and earlier in time series compared to males, but no overall difference in incidence in the two sexes. The factors significantly associated with HAUTI included: duration of hospitalization, per urethral catheterization and the duration of catheterization. *E. coli, Pseudomonas, Kebsiella*, and *Candida* accounted for over 90% of the isolates, and 73.5% of these were resistant to all the antibiotics for which sensitivity was tested. The remaining isolates demonstrated sensitivity to amikacin and/or cefoperazone-sulbactam.

**Conclusion:**

High infection rate coupled with widespread isolation polyantimicrobial resistant nosocomial pathogens emphasizes the importance of meticulous surveillance of nosocomial infections in the hospital, with due attention to antibiotic prescription practices.

## INTRODUCTION

Hospital acquired urinary tract infections (HAUTI) account for 35-45% of the nosocomial infections.[1–9] Apart from increasing the morbidity and emotional suffering among the patients, HAUTI escalates the medical care expenses in the form of prolonged hospital stay, lost work-days, laboratory costs, and the drug costs.[1,3] While most of the episodes are either asymptomatic or produce minor sequelae due to spread of infection to the contiguous organs, 2-4% of the cases may develop life threatening septicemia.[3,5,10] It is observed that 30-40% of the gram negative septicemia acquired in the hospital originates in the urinary tract.[11] Further, the in-patient deaths among the victims of HAUTI are 2 to 3-times higher than that among the non-bacteriuric patients.[12]

Variety of risk factors pertaining to aetiopathogenesis of HAUTI are identified,[3,5,6,10,13] according to which the control measures are instituted. The infection control measures in Indian hospitals are, however, constrained by the paucity of research in the field and the lack of hospital-specific data on nosocomial infections. Baseline estimates of the magnitude of the problem and the extent of antimicrobial resistance among the nosocomial pathogens are the minimum essential prerequisites for any hospital infection control programme. A study was, therefore, undertaken in the representative medicine and surgery wards of an apex medical college hospital in Goa to study the incidence and microbiological aspects of HAUTI, and also some of the patient-related factors associated with its occurrence.

## MATERIALS AND METHODS

A prospective study among 498 patients with minimum hospital stay of 48 h, from the randomly sampled medicine and surgery wards, was undertaken in an apex medical college hospital in Goa during July to December 2005. Patients less than 15 years of age were excluded. Baseline data was collected as regards to patient identification, age, sex, provisional diagnosis, treatment details, hemoglobin, blood sugar level, and serum creatinine. Urine examination for pus cells was performed on the day of admission, and on every alternate day thereafter. Demonstration of pyuria, i.e., more than 10 pus cells per cubic milliliter of urine was considered to be sufficient for the diagnosis of urinary tract infections (UTI).[14] Urine culture-sensitivity was done for those with pyuria for identification of the causative organism and its antimicrobial sensitivity using the Kirby-Bauer disc diffusion method. For those with positive culture reports, repeat culture were done weekly; isolation of the same organism(s) with the same antimicrobial sensitivity pattern was regarded as a single episode of HAUTI. All the isolates were counted once, irrespective of their isolation in pure or mixed cultures. Thus, the number of nosocomial isolates could be more than the episodes of HAUTI accounting for mixed cultures of more than one organism. However, the samples were considered to be contaminated on isolation of more than two organisms;[2] such samples were discarded (not included in the analysis), and the repeat samples were collected.

UTI was diagnosed as per the CDC definition[15] as follows-

Presence of at least two of the following with no other recognized cause: fever, urgency of urination, dysuria or suprapubic tenderness; with at least one of the following: pyuria or positive urine culture. Any episode of UTI that was not present in first 48 h of admission, and became apparent after 48 h of admission was diagnosed as HAUTI.

### Statistical Analysis

The data was analyzed using the SPSS for windows, version 10.5. Incidence of HAUTI is expressed as the infection rate (number of patients infected per 100 admissions), and as infection percentage (number of episodes of HAUTI per 100 admissions). Association of HAUTI with the factors under evaluation was studied by the application of the chi square test, the test of significance for the difference between the proportions and the unpaired *t* test for the difference between means, at 5% level of significance. Effect of potential confounding variable, wherever applicable, was controlled by stratification for the variable. Strength of the association was expressed as Odd's Ratio (OR), with 95% confidence interval calculated by Wolff's method.[16]

## DISCUSSION

Of the 498 patients, 40 developed 45 episodes of HAUTI. The overall infection rate was 8.03 per 100 admissions and the infection percentage was 9.03%. Only the first episode of HAUTI was considered in the analysis. All the infections were seen among the patients with indwelling per urethral catheter. The difference in the incidence of HAUTI among the males (18/274) and females (22/224) was not found to be statistically significant (χ^2^ = 1.765, *P*=0.184). More females were catheterized as compared to that of males, and this could account for higher incidence of HAUTI among females. The analysis was then restricted to catheterized males and females. Table 1 describes the age–gender distribution of HAUTI among the catheterized patients.

**Table 1:** Age-sex distribution of HAUTI*

Age-group	Gender						Total		
		
	Male			Female		
			
	Number	UTI+	Percent	Total	UTI+	Percent	Number	UTI+	Percent
12-25	5	0	0.00	8	2	25.0	13	2	15.4
26-35	16	1	6.25	19	5	55.6	35	6	17.1
36-45	12	6	50.0	11	3	27.3	23	9	39.1
46-55	13	5	38.5	10	1	10.0	23	6	26.1
56-65	7	4	57.1	6	5	83.3	13	9	69.2
66-75	4	2	50.0	4	3	75	8	5	62.5
>75	0	0	0.00	4	3	75	4	3	75.0
Total	57	18	31.6	62	22	35.5	119	40	33.6

* Only catheterized patients included

It was observed that the incidence of HAUTI was more among the catheterized females (22/62) compared to their male counterparts (18/57), but statistically not significant (χ^2^=0.203, *P*=0.652) implying no significant gender differences in the incidence of HAUTI. The finding is in strong disagreement with the findings of other researchers, that females have a stronger predilection for HAUTI compared to males.[1,3,12,13]

**Figure 1:**
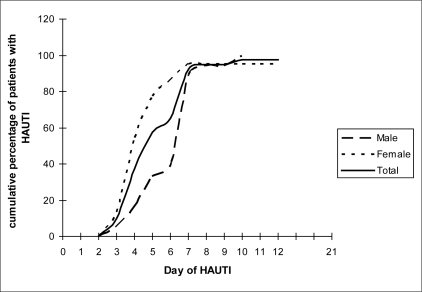
Occurrence of HAUTI among males and females according to its day of diagnosis

Figure 1 depicts the cumulative frequency curve of the patients developing HAUTI according to its day of diagnosis. It is observed that the catheterized females developed HAUTI earlier in time series (median=3.8 days) compared to the males (median=6.2 days). While 33.33% of the HAUTI among males developed within the first five days, 72.72% females developed it during the same time. The difference in the time of development of HAUTI among the males and the females was found to be statistically significant (χ^2^ =6.208, *P*=0.012). By the eighth day the proportion developing HAUTI was similar in both, but females developed it earlier than the males. These observations denote higher vulnerability of catheterized females to HAUTI, which could be attributed to shorter length of female urethra, it's proximity to anal canal, and absence of prostatic secretions in females. However, the finding of higher incidence among females as found by other researchers is not supported in this study.

HAUTI affected females at an earlier age than the males. While only 4.8% (1/21) of the catheterized males less than 35 years developed HAUTI, 25.9% (7/27) of the catheterized females less than 35 years developed it. This difference was found to be statistically significant (*P*<0.001). Similar observation concerning occurrence of HAUTI at an earlier age among females has been made by Turck and Stamm.[10]

Table 2 presents the analysis of some factors associated with the occurrence of HAUTI in the study population. A statistically significant association was noted as the incidence of HAUTI increased with the duration of hospitalization. The mean length of stay was for those with HAUTI was 12.02 (±9.08), compared to 6.27 (±4.16) for those without HAUTI. Thus, the victims of HAUTI spend an average of 5.75 extra hospital days compared to the non-sufferers (*t*=4.498, *P*=0.000). The association has been observed in the other studies as well[1,13] and could be expressed as the cause or the effect of HAUTI. While the prolonged hospital stay by itself could expose the patient to the hospital flora for a longer time, the seriously ill patients who are at risk of prolonged hospitalization may acquire HAUTI on account of their immunoincompetant status. Further, nosocomial infections, including HAUTI, are known to add to the morbidity by increasing the number of hospital bed-days.[17,18]

**Table 2:** Factors associated with hospital acquired urinary tract infections

Variables	N	HAUTI	^2^	*P*
Length of stay in days				
Less than 5	53	6 (11.3)	26.488	0.000
5 to 10	39	17 (43.6)		
10 to 15	15	8 (53.3)		
>15	12	9 (75.0)		
Sensorium				
Altered sensorium	62	18 (29.0)		>0.05
Conscious	57	22 (38.6)		
Outcome of admission				
Non-fatal	71	22 (31.0)		>0.05
Death	48	18 (37.5)		
Duration catheter in days				
Less than 4 days	47	3 (6.4)	39.746	0.000
4 to 7	41	14 (34.1)		
8 to 14	22	15 (68.2)		
More than 14	9	8 (88.9)		
Total	119	40 (33.6)		

Figures in the parentheses are in percentage

The patients with altered sensorium were at a higher risk, though not statistically significant (*P*>0.05), of HAUTI. Altered sensorium expresses the severity of the underlying illness and also the need for per urethral catheterization among the patients, both of which are, independently, the risk factors for HAUTI.

The victims of HAUTI are more at risk of fatal outcome, OR= 1.34 (0.83-1.7), i.e., 34% higher mortality compared to the ones without HAUTI. The association was, however, not statistically significant (*P*>0.05). Excess mortality among the bacteriuric patients has been observed by the other researchers worldwide[12,19] and is attributed to life threatening complications of HAUTI, like septicemia[3,5,10,11] or the severity of underlying illness which by itself predisposes the patient to HAUTI.

Per urethral catheter is identified, worldwide, as the single most important predisposing factor for the HAUTI.[1–3,13,19] The catheter itself, if improperly maintained, may serve as a portal of entry for the pathogens. The pathogens may gain entry at the time of catheter insertion, or later by intraluminal or transurethral spread.[5] Further, the aetiopathopgenic importance of underlying illness that necessitated catheterization may not be ignored. All the episodes of HAUTI in the study were among the catheterized patients. Further, as shown in Table 2, a statistically significant association was noted between the duration of catheterization and the incidence of HAUTI (*P*=0.000). The mean duration of catheterization among the patients with HAUTI was 10.3 (±6.02) as against 4.37 (±3.22) in those without HAUTI. This was a statistically significant association (*t*=7.017, *P*=0.000) indicating that the risk of HAUTI increased with the increasing duration of catheterization.

Table 3 represents the microbiologic characteristics of HAUTI. In all 55 isolates were obtained from the 45 episodes of HAUTI, accounting for 10 episodes of mixed growth (maximum up to 2 isolates). The episodes with multiple isolates were traced to those with long-standing indwelling catheter of more than 10 days. The finding correlates well with the opinion of Burke *et al*,[20] who state that - a single infection species is responsible for 80% of the HAUTIs in the patients with short-term catheters; but most patients with long-term catheters have polymicrobial infection.

**Table 3:** Microbiology and antimicrobial sensitivity of HAUTI*

Organisms	Total	Resistant to all	Sensitive†
	
	No. (%)	No. (%)	No. (%)
Escherichia coli	27 (49.1)	22 (81.5)	5 (18.5)
Pseudomonas aeruginosa	7 (12.72)	5 (71.4)	2 (28.6)
Klebsiella	7 (12.72)	4 (57.1)	3 (42.9)
Candida albicans	6 (10.91)	-	-
Aceinetobacter baumanii	3 (5.46)	3 (100)	0
Others	5 (9.1)	2 (40)	3 (60)
Total	55 (100)	36 (73.5)	13 (26.5)

* Antimicrobial sensitivity testing was not done for *Candida*

† 3 sensitive to amikacin only, 5 to magnex only, and 5 to both Others include 2 isolates of *Citrobacter freundii*, and one each of *A. colcoaceticus, S. pyogenes and C. diversus*

Gram negative organisms were the most frequent isolates, with *E. coli* being the most common followed by *Pseudomonas and Klebsiella. Candida albicans* accounted for almost 11% of the organisms, and was isolated in pure cultures in all the instances. Only one of the candidal UTIs was the first episode of HAUTI, the rest 5 being the second episode among those who already had HAUTI in the preceding week of hospitalization. All the candidal UTIs were encountered among the critically ill (uraemic encephalopathy, viral encephalitis, bacterial peritonitis, diabetic ketoacidosis, stroke, and alcoholic liver disease with septicemia), 50-plus aged patients with the mean duration of catheterization of 14.3 days (SD 4.23), and all of which had fatal outcome of hospitalization. The frequency of candidal infections is comparable to that in other studies.[21,22] In addition, there is an ample of evidence to believe that *Candida albicans*, though a normal genital flora, becomes pathogenic amidst factors like surgery, total parenteral nutrition, broad spectrum antibiotics, and indwelling catheter; and that it has a significant impact on the outcome of hospitalization.[22,23]

The antimicrobial sensitivity was tested for the following antibiotics: amoxycillin, augmentin (amoxycillin with clavulinic acid), tetracycline, co-trimoxazole, roxithromycin, azithromycin, oxacillin, chloremphenicol, amikacin, gentamicin, tobramycin, netromycin, carbenicillin, teicoplenin, cefadroxyl, cefuroxime, cefoperazone, magnex (cefoperazone with sulbactam), ceftriaxone, cefotaxime, ceftizoxime, ceftazidime, nalidixic acid, norfloxacin, ciprofloxacin, furazolidone, rifampicin, vancomycin, and levofloxacin. A high degree of antimicrobial resistance was noticed with almost 73% of the isolates being resistant to all the antibiotics for which tested. The rest of the isolates demonstrated *in vitro* sensitivity to magnex and/or amikacin.

A clinically significant correlation was found between the antibiotic usage in the study wards and the resistance pattern. Systemic metronidazole, penicillins, fluoroquinolones, and ceftriaxone were administered in, respectively, 32%, 25%, 23%, and 17% of the study patients, and none of the nosocomial isolates were sensitive to any of these. On the other hand, 16% of the isolates were sensitive to amikacin and 20% to magnex, which were administered to, respectively, 2% and 1% of the patients. The issue is elaborated by Kamat *et al*,[24] in the research article on nosocomial antimicrobial resistance. The findings are in agreement with the other researchers worldwide[8,25] and emphasizes the role of selective drug pressure in emergence of drug resistant mutants. Sensitivity to amikacin and magnex amidst widespread polyantimicrobial resistance is supported by the similar studies by Prashant *et al*[26] and Gupta.[27]

## CONCLUSION

The paper, while identifying the various factors associated with the occurrence of HAUTI, emphasizes on the issue of sex differential in its occurrence. Isolation of polyantimicrobial resistant species of nosocomial pathogens and its relation to the antibiotic prescription in the study wards calls for routine surveillance of nosocomial infections in the hospital, coupled with the institution of evidence-based antibiotic prescription policy.

## References

[1] Foxman B (2002). Epidemiology of urinary tract infections: Incidence, morbidity and economic costs. Am J Med.

[2] Ducel G, Fabry J, Nicolle L, WHO (2002). Epidemiology of nosocomial infections. Prevention of hospital acquired infections: A practical guide.

[3] Stamm WE, Benett JV, Brachman PS (1998). Urinary tract infections. Hospital infections.

[4] Dillman C (1996). Epidemiology of nosocomial infections: 10-month experience in one hospital. Curr Ther Res.

[5] Stamm WE (1991). Catheter associated urinary tract infections: Epidemiology, pathogenesis and prevention. Am J Med.

[6] Garibaldi RA, Burke JP, Dickman ML, Smith CB (1974). Factors predisposing to Bacteriuria during indwelling urethral catheterization. N Engl J Med.

[7] Eickhoff TC, Brachman PS, Bennet JV, Brown JF (1969). Surveillance of nosocomial infections in community hospitals: Surveillance methods, effectiveness and initial results. J Infect Dis.

[8] Barrett FF, Casey J, Finland M (1968). Infections and Antibiotic use among patients at Boston city hospital, February 1967. N Engl J Med.

[9] Thoburn R, Robert F, Cluff LE, Virginia BM (1968). Infections acquired by hospitalized patients: An analysis of the overall problem. Arch Intern Med.

[10] Turck M, Stamm WE (1981). Nosocomial infection of the urinary tract. Am J Med.

[11] Stamm WE (1975). Guidelines for prevention of catheter associated urinary tract infections. Ann Intern Med.

[12] Platt R, Polk F, Murdock B, Rosner B (1982). Mortality associated with nosocomial urinary-tract infection. N Engl J Med.

[13] Graves N, Tong E, Morton AP, Halton K, Curtis M, Lairson D (2007). Factors associated with health care-acquired urinary tract infection. Am J Infect Control.

[14] Stamm WE (1983). Measurement of pyuria and its relation to bacteriuria. Am J Med.

[15] Gaynes RP, Horan TC, Mayhall GC (1999). Surveillance of nosocomial infections. Hospital epidemiology and infection control.

[16] Ajay Kumar MV (2006). Confidence Intervals and tests of Significance. Indian J Community Med.

[17] Freeman J, Rosner BA, McGowan JE (1979). Adverse effects of nosocomial infection. J Infect Dis.

[18] Haley RW, Schaberg DR, Crossley KB, Von Allmen SD, McGowan JE (1981). Extra charges and prolongation of stay attributable to nosocomial infections: A prospective interhospital comparison. Am J Med.

[19] Mehta A, Rosenthal VD, Mehta Y, Chakravarthy M, Todi SK, Sen N (2007). Device-associated nosocomial infection rates in intensive care units of seven Indian cities: Findings of the International Nosocomial Infection Control Consortium (INICC). J Hosp Infect.

[20] Burke JP, Zavasky DM, Mayhall GC (1999). Nosocomial urinary tract infection. Hospital epidemiology and infection control.

[21] Gelman J, Rajfer J, Massry SG, Glassock (2001). Use of the urethral catheter. Textbook of nephrology.

[22] McNeil MM, Mayhall GC (1999). Hospital epidemiology and infection control.

[23] Rubin RH, Tolkoff-Rubin NE, Cotrans RS, Brenner BM, Rector FC (1996). Urinary tract infection, pyelonephritis and reflux nephropathy. The kidney.

[24] Kamat US, Fereirra AM, Savio R, Motghare DD (2008). Antimicrobial resistance among nosocomial isolates in a teaching hospital in Goa. Indian J Community Med.

[25] Eickhoff TC, Benett JV, Brachman PS (1998). Antibiotics and nosocomial infections. Hospital infections.

[26] Prashanth K, Badrinath S (2004). *Invitro* susceptibility pattern of aceinetobacter species to commonly used cephalosporins, quinolones and aminoglycosides. Indian J Med Microbiol.

[27] Gupta SK (2000). Cefoperazone-Sulbactam in surgical infections. Hosp Today.

